# First molecular confirmation of the presence of *Hippobosca longipennis* (Diptera: Hippoboscidae) and infestation of sheltered dogs in Morocco

**DOI:** 10.1186/s13071-025-06830-y

**Published:** 2025-05-27

**Authors:** Maria Bourquia, Abderrahmane Zahri, Mehdi Ahlamine, Thomas Balenghien, Paula Meyer, Felix Gregor Sauer, Renke Lühken

**Affiliations:** 1 Parasitology and Parasitic Diseases Unit, Department of Animal Pathology and Public Health, Hassan II Institute of Agronomy and Veterinary Medicine, Rabat, Morocco; 2 CIRAD, UMR ASTRE, 34398 Montpellier, France; 3 ASTRE, University of Montpellier, CIRAD, INRAE, Montpellier, France; 4 Bernhard Nocht Institute for Tropical Medicine, Bernhard‑Nocht‑Str. 74, 20359 Hamburg, Germany

**Keywords:** *Hippobosca longipennis*, Dog louse fly, Molecular barcoding, Dogs, Morocco, Nematodes

## Abstract

**Background:**

*Hippobosca longipennis* (Diptera: Hippoboscidae) is an obligate hematophagous ectoparasite that infests a wide range of vertebrate hosts across Africa, Southern Europe, the Middle East, and Asia. It is a potential vector of *Acanthocheilonema dracunculoides* (Filarioidea: Onchocercidae) and serves as a phoretic host for *Cheyletiella yasguri* (Acari: Cheyletiellidae), a known causative agent of dermatitis in both dogs and humans. Due to the lack of data on hippoboscids in Morocco, this study aimed to investigate the louse fly fauna of sheltered dogs in the country as well as the filarial nematodes they may harbor.

**Methods:**

Between April and November 2022, 230 sheltered dogs from four cities in Central Morocco were randomly examined as part of an entomological and epidemiological study on arthropod vectors and canine vector-borne pathogens. All visible louse flies on the domestic dogs were randomly collected and then morphologically and molecularly identified. DNA was subsequently extracted for screening of filarial nematodes.

**Results:**

A total of 30 dogs (13.1%) were infested with 35 *H. longipennis* louse flies, consisting of 33 adults (10 males, 19 non-gravid females, and four gravid females) and two larvae. Two representative specimens were confirmed through DNA barcoding of the cytochrome oxidase subunit I gene. All fly pools (gravid females, non-gravid females, males, and larvae) tested negative for filarial nematodes in the 12S rRNA PCR.

**Conclusions:**

This study represents the first morphological and molecular characterization of *H. longipennis* flies in Morocco. Further national-scale investigations are needed to address gaps in the knowledge of unrecorded hippoboscid species and the pathogens of medical and veterinary importance that they may carry.

**Graphical Abstract:**

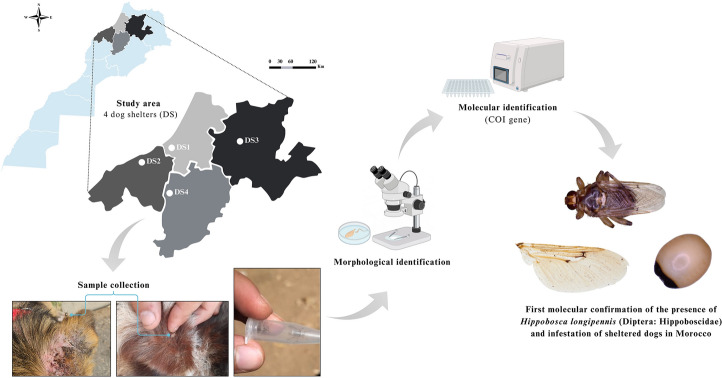

**Supplementary Information:**

The online version contains supplementary material available at 10.1186/s13071-025-06830-y.

## Background

*Hippobosca longipennis* (Fabricius, 1805), commonly known as the dog louse fly (Diptera: Hippoboscidae), is a blood-feeding ectoparasite that affects a wide variety of hosts. These include domestic dogs (*Canis familiaris*) and cats (*Felis catus*) as well as wild animals such as foxes (*Vulpes vulpes*), jackals (*Canis aureus*), leopards (*Panthera pardus*), lions (*Panthera leo*), cheetahs (*Acinonyx jubatus*), mongooses (*Urva auropunctata*), civets (*Paguma larvata*), hyenas (*Crocuta crocuta*), birds (*Columba livia domestica*), and antelopes (*Pantholops hodgsonii*) [1]. This species is also responsible for occasional human nuisance [2, 3]. *Hippobosca longipennis* has been discovered in mummified dogs in Egypt [4] and is native to the eastern and northern regions of Africa as well as the Middle East [2]. Initially, the species was primarily associated with domestic dogs and wild canids; however, its global distribution has expanded significantly due to globalization, now including Europe, Asia, and North America [2]. In the USA, the first recorded sighting of *H. longipennis* was in San Diego, California, in 1970, likely imported via infested cheetahs [5]. Since then, the species has been reported in various states, including Oregon, Georgia, and Texas [6]. In India, *H. longipennis* has been collected from street dogs [7]; recently, the species has also been observed on domestic dogs and road-killed wild cats in Romania [8]. Additionally, fly infestations caused by *H. longipennis* are widespread in domestic dogs across several European countries, especially in the Mediterranean region [2, 9, 10].

*Hippobosca longipennis* is a potential vector of *Acanthocheilonema dracunculoides* (Filarioidea: Onchocercidae) [11], a filarial nematode that has been reported in dogs across Morocco, Algeria, Spain, Portugal, and Italy [12–16]. It has also been documented in foxes in Italy [17]. Additionally, *Rhipicephalus sanguineus* sensu lato ticks are recognized as important vectors of this canid filarial worm [18, 19]. *Hippobosca longipennis* is also known to serve as a transport host for various mite species, including *Cheyletiella yasguri* (Acari: Cheyletiellidae), which is a well-established cause of *Cheyletiella* dermatitis in both dogs and humans [7, 20]. The presence of *H. longipennis* in certain Moroccan cities was first documented years ago in Meknès [21] and Marrakech [22]. However, until the present study, the presence of *Hippobosca* species in the dog population of Morocco had, to our knowledge, never been molecularly confirmed or systematically monitored. Therefore, we conducted a comprehensive survey of sheltered dogs across the country to identify the fly species using morphological analysis and DNA barcoding while also performing molecular screening for filarial nematodes through specific PCR techniques.

## Methods

**Fig. 1:**
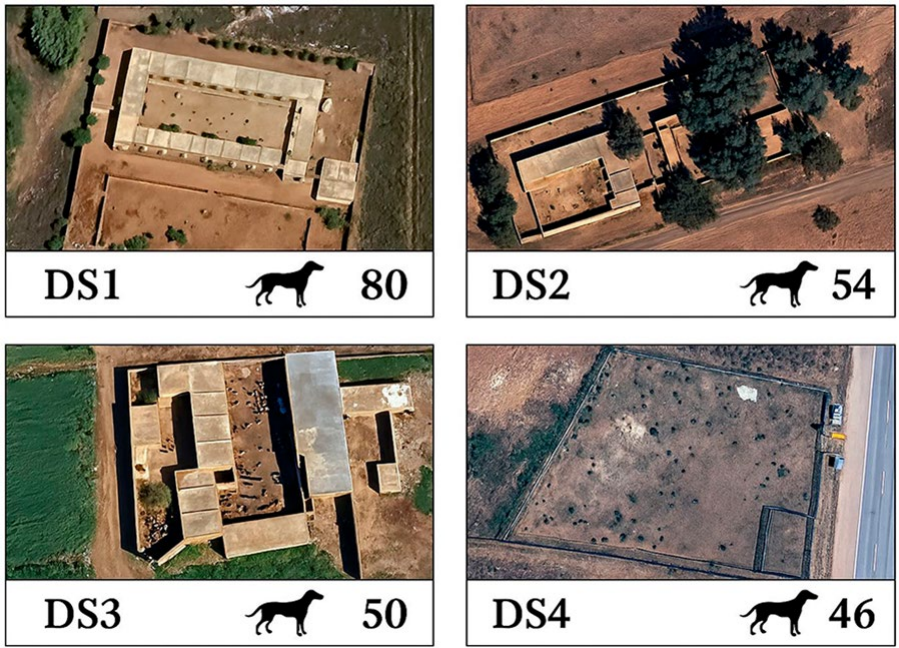
Satellite images of the four shelters (DS) along with the number of dogs at each location

**Fig. 2:**
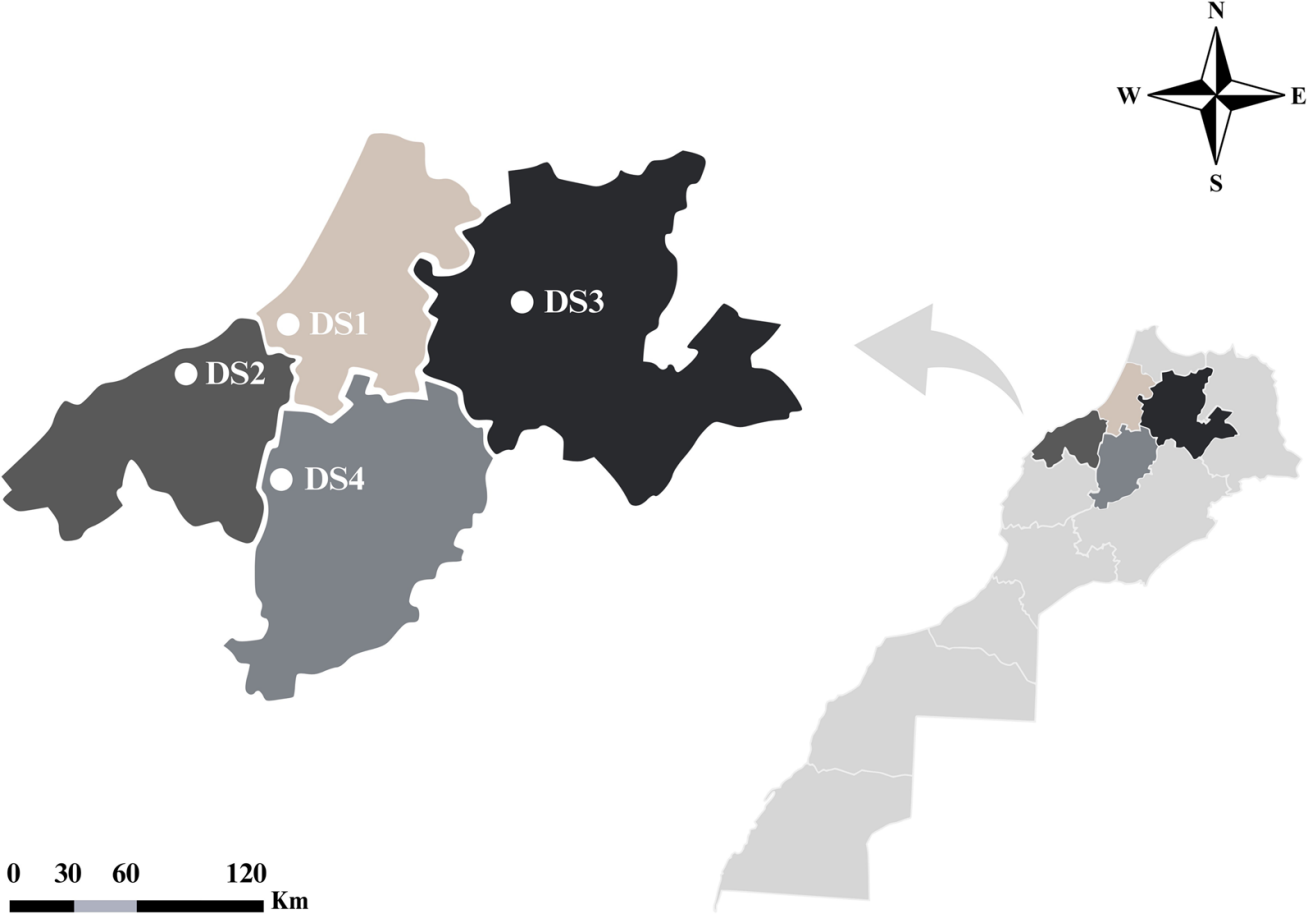
Moroccan map highlighting the localization of the four dog shelters (DS)

From April to November 2022, a total of 230 dogs were randomly selected and individually examined for ticks at four private and licensed dog shelters (DS) across Central Morocco as part of an entomological and epidemiological study on arthropod vectors and canine vector-borne pathogens. The shelters were located in Rabat (DS1; 33.806538 N, 6.902472 W; *number of dogs* = 80), Casablanca (DS2; 33.507278 N, 7.631269 W; *n* = 54), Fez (DS3; 34.004291 N, 5.106422 W;* n* = 50), and Khouribga (DS4; 32.845631 N, 6.938536 W; *n* = 46) (Figs. 1and 2). Visible louse flies were collected from each infested dog following thorough manual and visual inspections. The collected flies were carefully transported to the Parasitology and Parasitic Diseases laboratory at the Hassan II Institute of Agronomy and Veterinary Medicine in Rabat (Morocco). Each specimen was categorized by developmental stage (larva, adult, gravid and non-gravid females) and sex (male and female) and identified morphologically based on distinct features of the head, thorax, legs, claws, and wings, as outlined by Bequaert [23]. Biological samples were stored at − 80 °C before being shipped to the Bernhard Nocht Institute for Tropical Medicine in Hamburg, Germany, for molecular species identification and the molecular detection of filarial nematodes.

To confirm the fly species at the molecular level, DNA extraction was performed on the legs of two selected specimens (one male and one non-gravid female) using a QIAamp DNA Mini Kit (Qiagen GmbH, Hilden, Germany). The random selection of these specimens was based on the identical morphological features shared by all the collected individuals. A 709-bp fragment of the mitochondrial cytochrome c oxidase subunit I (COI) gene was amplified using universal primers as described by Folmer et al. [24]. The PCR protocol consisted of an initial denaturation step at 95 °C for 15 min, followed by 40 cycles of amplification (94 °C for 30 s, 54 °C for 45 s, and 72 °C for 1 min), and a final extension at 72 °C for 10 min. The resulting amplicons were processed by Sanger sequencing (LGC Genomics, Berlin, Germany), with sequences pre-processed using Geneious^®^ 7.1.9 [25]. The final sequences were then compared against GenBank sequences (http://blast.ncbi.nlm.nih.gov/Blast.cgi). The louse flies were categorized into four groups: gravid females, non-gravid females, males, and larvae. Each group underwent DNA extraction and screening for filarial nematodes. To prepare the pools, 180 μl of buffer ATL (DNeasy Blood & Tissue Kits, Qiagen) was added to 1.5-ml Eppendorf tubes, along with two 1.4-mm ceramic beads (Qiagen, Hilden, Germany). The samples were then homogenized for 6 min at room temperature using a TissueLyser II (Qiagen, Hilden, Germany). Next, 20 μl of proteinase K was added to each pool, followed by vortexing and overnight incubation at 56 °C. DNA extraction was carried out using the QIAGEN DNeasy Blood & Tissue Kit (QIAGEN, Hilden, Germany), strictly following the manufacturer’s protocol. The extracted DNA was then screened for filarial nematodes through conventional PCR, targeting the 12S ribosomal RNA and mitochondrial cytochrome c oxidase subunit I (COI) genes, as per established protocols [26, 27]. DNA of *Acanthocheilonema dracunculoides* served as the positive control, with distilled water used as the negative control in all PCR assays.

## Results

**Fig. 3:**
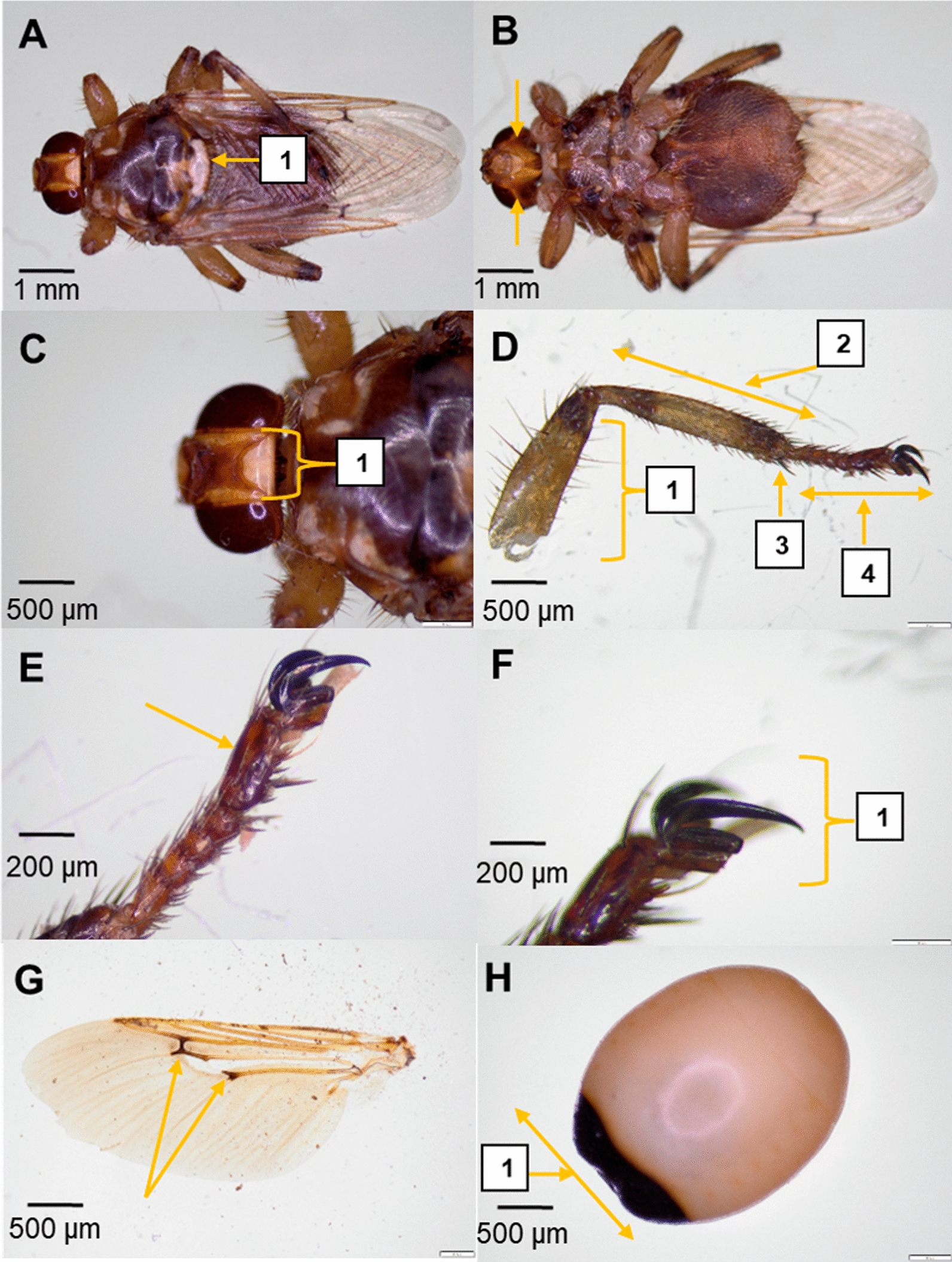
*Hippobosca longipennis*, the dog louse fly. **A** Dorsal view of winged gravid female (1 scutellum is white). **B** Ventral view of a winged gravid female with characteristic triangular brown spots on the ventral view of the head (arrows). **C** Dorsal view of the head (1: sharp and triangular apical lobes of the fronto-clypeus with straight internal margins). **D** Foreleg (1: fore, mid, and hind part of the femur covered by few long and short setae; 2: a few short setae characterize the fore and mid part of the tibia; 3: hind tibia with bristle-like long setae; 4: tarsal claws). **E** Tarsal segment covered by a row of short setae with longer tarsus 5 (arrow) compared to tarsus 1, 2, 3, and 4. **F** Tarsal claws (1: asymmetrical claw bifurcation). **G** Left wing of a male, developed and comprising pale veins with dark spots (arrows). **H** Third-stage larva (L3) (1: non-segmented structure with a dark stigmatic plate on the posterior side)

Of 230 dogs sampled, 30 (13.1%) were infested with *H. longipennis* (Fig. 3). The infested dogs included 11 males and 19 females, ranging in age from 1 to 7 years. All were mongrels, except for one Siberian husky (Additional file: Table S1). A total of 35 flies were collected between June and September 2022, of which 33 (94.3%) were adults (10 males, 19 non-gravid females, and four gravid females) and two were larvae (L3). The flies were collected from the neck and back of dogs at a single shelter in Khouribga (DS4); no louse flies were found on dogs at the other three shelters (DS1, DS2, DS3). Molecular barcoding of the COI gene from two representative louse fly specimens confirmed the morphological identification of *H. longipennis*. The sequences were submitted to GenBank (accession nos. PQ877319 and PQ877320). All fly pools tested negative for nematodes using 12S rDNA [26] and cytochrome c oxidase [27] PCR assays.

## Discussion

In this study, the dog louse fly, *Hippobosca longipennis*, is described and molecularly confirmed for the first time to our knowledge in domestic dogs in Morocco. *Hippobosca longipennis* is a widespread hematophagous ectoparasite of both domestic and wild carnivores found in the Middle East, North Africa, and East Africa [2, 28]. The prevalence of fly infestation detected in this study (13.1%) is consistent with previous reports from two studies in domestic dogs in Egypt (18%, [29]; 16%, [30]), and *H. longipennis* has also been widely reported in dogs from Kenya [31] and Iran [32]. Additionally, this species has been recorded in wild carnivores in Europe, including genets (*Genetta genetta*) [33], 31% of endangered Iberian lynx (*Lynx pardinus*), and 26% of red foxes (*Vulpes vulpes*) in southern Spain [34], as well as in a young male red fox parasitized by 14 *H. longipennis* in Turkey [35]. Interestingly, larvae of *H. longipennis* were found on mongrel dogs at DS4 but not at the other three shelters. Larviposition at this shelter may have occurred in the soil or within cracks in the walls, which provided hiding places for these immature stages [1, 36]. These larvae were likely carried by the wind and inadvertently transferred to the dogs. The absence of flies at DS2 can be attributed to insecticide treatments, while their exclusive presence at DS4 suggests that optimal biotic and abiotic conditions there influence their abundance compared to DS1 and DS3 [37].

*Hippobosca longipennis* is a known vector of *A. dracunculoides* [11]. Additionally, ten of 65 *H. longipennis* flies collected from 101 dogs in Northern India were found to harbor up to 12 infective larvae of a potential new *Acantocheilonema* species [7]. However, there is no definitive evidence that these flies can transmit this potentially new nematode [7]. The negative PCR results for filarial nematode detection in the collected flies suggest that the pathogen is not circulating in the area, which remains clinically silent [38]. Future studies with larger sample sizes should be conducted to verify this hypothesis.

## Conclusions

Given the limited research on hippoboscid flies in Morocco, large-scale entomological surveys are essential to better characterize the species composition and to further investigate the distribution of the vector-borne pathogens they may carry across different regions of the country.

## Supplementary Information

Additional file 1: Table S1. Data on louse flies collected from sheltered dogs in Morocco.

## Data Availability

No datasets were generated or analysed during the current study.

## Abbreviations

COI: Cytochrome oxidase subunit I
DS: Dog shelter
PCR: Polymerase chain reaction
