# Supplementary material for: First molecular confirmation of the presence of Hippobosca longipennis (Diptera: Hippoboscidae) and infestation of sheltered dogs in Morocco
Source: Parasit Vectors. 2025 May 27;18:193. doi: 10.1186/s13071-025-06830-y (PMC12107861; doi:10.1186/s13071-025-06830-y)
Supplement: Supplementary file 1 — Additional file 1: Table S1. Data on louse flies collected from sheltered dogs in Morocco. [file 13071_2025_6830_MOESM1_ESM.docx]

**Additional file: Table S1.** Data on louse flies collected from sheltered dogs in Morocco.

| **Sampling**  **date** | **Locality** | **WGS84-y** | **WGS84-x** | **Dog breed** | **Sex** | **Age (years)** | ***Hippobosca***  ***longipennis*** |
| --- | --- | --- | --- | --- | --- | --- | --- |
| 26-06-2022 | Khouribga | 32.845631 | 6.938536 | Mongrel | F | 3 | 1 GF + 1 L |
| 26-06-2022 | Khouribga | 32.845631 | 6.938536 | Mongrel | M | 4 | 1 M |
| 26-06-2022 | Khouribga | 32.845631 | 6.938536 | Mongrel | F | 3 | 1 NGF |
| 27-06-2022 | Khouribga | 32.845631 | 6.938536 | Mongrel | M | 5 | 1 NGF |
| 27-06-2022 | Khouribga | 32.845631 | 6.938536 | Mongrel | M | 2 | 1 M |
| 26-07-2022 | Khouribga | 32.845631 | 6.938536 | Mongrel | F | 2 | 1 NGF |
| 26-07-2022 | Khouribga | 32.845631 | 6.938536 | Mongrel | F | 1 | 1 NGF |
| 26-07-2022 | Khouribga | 32.845631 | 6.938536 | Mongrel | F | 3 | 1 M |
| 26-07-2022 | Khouribga | 32.845631 | 6.938536 | Mongrel | F | 4 | 1 M |
| 27-07-2022 | Khouribga | 32.845631 | 6.938536 | Mongrel | F | 4 | 1 M |
| 27-07-2022 | Khouribga | 32.845631 | 6.938536 | Mongrel | F | 2 | 1 M |
| 27-07-2022 | Khouribga | 32.845631 | 6.938536 | Mongrel | F | 1 | 1 NGF |
| 27-07-2022 | Khouribga | 32.845631 | 6.938536 | Mongrel | M | 1 | 1 NGF |
| 27-07-2022 | Khouribga | 32.845631 | 6.938536 | Mongrel | F | 1 | 1 NGF |
| 26-08-2022 | Khouribga | 32.845631 | 6.938536 | Mongrel | F | 3 | 1 GF |
| 27-08-2022 | Khouribga | 32.845631 | 6.938536 | Mongrel | M | 4 | 1 NGF |
| 27-08-2022 | Khouribga | 32.845631 | 6.938536 | Mongrel | F | 2 | 1 NGF |
| 27-08-2022 | Khouribga | 32.845631 | 6.938536 | Mongrel | F | 1 | 1 NGF |
| 27-08-2022 | Khouribga | 32.845631 | 6.938536 | Siberian Husky | F | 2 | 2 M + 3 NGF |
| 26-09-2022 | Khouribga | 32.845631 | 6.938536 | Mongrel | M | 3 | 1 M |
| 26-09-2022 | Khouribga | 32.845631 | 6.938536 | Mongrel | M | 3 | 1 M |
| 26-09-2022 | Khouribga | 32.845631 | 6.938536 | Mongrel | F | 7 | 1 NGF |
| 26-09-2022 | Khouribga | 32.845631 | 6.938536 | Mongrel | F | 2 | 1 GF |
| 26-09-2022 | Khouribga | 32.845631 | 6.938536 | Mongrel | M | 1 | 1 NGF |
| 26-09-2022 | Khouribga | 32.845631 | 6.938536 | Mongrel | M | 1 | 1 NGF |
| 27-09-2022 | Khouribga | 32.845631 | 6.938536 | Mongrel | F | 3 | 1 GF |
| 27-09-2022 | Khouribga | 32.845631 | 6.938536 | Mongrel | M | 5 | 1 NGF |
| 27-09-2022 | Khouribga | 32.845631 | 6.938536 | Mongrel | F | 4 | 1 NGF |
| 27-09-2022 | Khouribga | 32.845631 | 6.938536 | Mongrel | F | 3 | 1 NGF |
| 27-09-2022 | Khouribga | 32.845631 | 6.938536 | Mongrel | M | 2 | 1 L |
| **Total** | | | | | | | **35** |

GF = Gravid Female; M = Male; NGF = Non-Gravid Female; L = Larva
